# Transforming Public Health Through Community Partnerships

**Published:** 2005-10-15

**Authors:** Neil E. Hann

**Affiliations:** Community Development Service, Oklahoma State Department of Health

This special issue of *Preventing Chronic Disease* highlights health education as a core function of public health. Health education is "an innate aspect of public health practice" as described by Lynne Wilcox in her editorial (1), and seven key areas of responsibilities for health educators serve as their fundamental competency base. These seven core areas include the following (2):

- Assessing individual and community needs for health education
- Planning effective health education programs
- Implementing health education programs
- Evaluating the effectiveness of health education programs
- Coordinating the provision of health education services
- Acting as a resource person in health education
- Communicating health and health education needs, concerns and resources (2)

How these seven areas of responsibility are implemented by health educators to achieve lasting behavior change or sustained community health improvement varies tremendously according to individual, family, and community needs. However, in recent years, it has become increasingly clear that the seven areas of health educator responsibilities are often effectively achieved through collaborative, community-partnership settings. This editorial describes the experience in transforming public health in Oklahoma and achieving successful health education and health promotion initiatives through community partnerships.

## From poor health outcomes to community partnerships

Oklahoma has had the unfortunate distinction of consistently ranking toward the bottom of national health rankings (3). Despite efforts to reverse these trends during the mid-1980s and through the 1990s, health status indicators in Oklahoma failed to move in a significantly positive direction. In fact, Oklahoma has been the only state since 1988 in which age-adjusted death rates have actually increased (4).

Clearly, this caused a great deal of concern among Oklahoma's health leaders, and innovative solutions were sought to reverse these negative trends. In 1997, an opportunity became available from The Robert Wood Johnson Foundation and the W. K. Kellogg Foundation. Called Turning Point, the program issued a request for proposals that encouraged local and state applicants to rethink the delivery of public health, placing emphasis on state and local collaborative partnerships and eliciting ideas on intervention priorities from community partners. Although implementation of these new approaches would represent a radical change in how public health would be delivered in Oklahoma, the state health commissioner at the time, Dr. Jerry R. Nida, decided to move forward with the Turning Point application because he understood the urgency of needing to change and restructure how public health was delivered in Oklahoma. The application, submitted by the Oklahoma State Department of Health (OSDH) and three community partnerships in Cherokee, Texas, and Tulsa counties in July 1997, included the following opening paragraph:

"Healthy Communities" is our vision for Oklahoma in the 21st century. In order to achieve this vision, work must begin now to change the health culture in Oklahoma through state and local partnerships. . . . [W]e must find innovative ways of working together, taking risks, in order to achieve our shared vision of healthy communities. These risks include questioning the business of health in Oklahoma as well as losing the comfort of predictability. . . . [W]e begin a new working dialogue in Oklahoma, in which community partners engage in a stronger leadership role and state partners assume a stronger technical resource position (5).

In January 1998, the OSDH was awarded a 2-year Turning Point planning grant of $300,000, and the three community partnerships were awarded $60,000 each for 3 years. During that period, models for transforming public health through community partnerships were developed in the three pilot sites, and the philosophy of the state partnering with communities for health improvement needs began to take shape. Each model proved successful in assessing local needs, establishing priorities, and implementing interventions tailored to the unique characteristics of the community. In January 2000, Oklahoma was awarded a 4-year grant of $950,000 to implement the Oklahoma Turning Point models on a statewide basis.

## Historical perspective

To understand the significance of Turning Point for Oklahoma, one must look at the history of public health infrastructure in Oklahoma and the transformations that are beginning to occur. Public health in Oklahoma has evolved into a centralized system, largely as a result of historical actions of the state legislature and categorical funding through federal sources. The central office of the OSDH, located in Oklahoma City, has traditionally directed public health decisions for Oklahoma. Although the centralized system has resulted in some positive outcomes, including a comprehensive bricks-and-mortar infrastructure with county health departments in 69 of 77 counties in Oklahoma, significant improvements in health status indicators have not been realized.

The lack of improvement in health, despite a good physical public health infrastructure and a well-trained workforce, has been an area of tremendous concern for the state board of health and others in the health field. When we examined the possible reasons for the lack of improvement in health, we saw clearly that the missing element was direct involvement of communities in public health decisions. Before Turning Point, decisions about public health were made at the central office and delivered in a cookie-cutter fashion for each county. Such a delivery system resulted in little progress toward local health improvement — each community has its own unique challenges, and the same approaches will not necessarily work in every area of the state. Unless communities are actively engaged in determining their own public health needs and developing and implementing solutions, improvement in community health will not be realized.

The key objective for the Oklahoma Turning Point initiative was to expand community health improvement partnerships into each county in Oklahoma using models developed in three original pilot Turning Point partnerships in Cherokee, Texas, and Tulsa counties.

## Key challenges and lessons learned

The key challenge for the Oklahoma Turning Point initiative has been providing enough skilled health department staff support to the community partnerships to ensure their success. Skilled staff support is even more important than having funds directly available for the partnerships. Regional health department field consultants have provided technical assistance in such areas as identifying priorities through data analysis, planning and implementing interventions based on priorities, and evaluating success. In addition, health department field consultants have provided assistance in such basic areas as developing partnership bylaws, conducting efficient and productive meetings, developing meeting agendas, recording partnership decisions through minutes, and communicating partnership activities through the local news media. Regional Turning Point health department field consultants are critical for each of these areas, and partnerships in Oklahoma have been successful because of the support provided by field consultants.

Other challenges that were encountered early but dealt with effectively were challenges common to most partnerships — turf and control issues. The willingness of OSDH to relinquish control and concern about who got credit for accomplishing health improvement efforts quickly nullified turf and control issues and allowed the Turning Point collaborative philosophy to flourish.

Through the Turning Point initiative, three key lessons on community health improvement partnerships were learned:

### Collaboration works

Without question, collaborative efforts to improve health are essential. Working together, sharing resources, and combining talents enhance the opportunities and likelihood for achieving positive health outcomes. Because of the complexity and cost of today's health environment, public health agencies and others involved in prevention efforts cannot afford to work in isolation. Collaboration results in positive outcomes that are superior to outcomes that result from agencies and organizations working separately on parallel paths.

### Giving up control and concern about who gets credit contributes to the success of partnerships

For collaboration to be successful, partners have to agree to give up complete control. Although one agency or organization in a collaborative effort may take a leadership position, all partners are equal, and it is the partnership that gets credit for success, not any one organization. Once all partners understand this concept, the partnership will thrive.

### Dedicated staff for partnership development is essential

As described earlier, regional skilled health department field consultants, who provided technical assistance and support, were key to the success of Turning Point in Oklahoma. All of the volunteers in the Oklahoma Turning Point partnerships have full-time jobs and responsibilities. Even when volunteer partners are completely dedicated and believe in the partnership philosophy to improve health outcomes, it is still difficult for a partnership to thrive without dedicated, paid staff support from a health department or another participating agency.

## System changes

The success in establishing partnerships across the state — and just as important, the success in ensuring the sustainability of the partnerships — has been better than the most hopeful expectations. There are now 50 partnerships based on the three original models in Cherokee, Texas, and Tulsa counties. The partnerships are in varying stages of development, with several new partnerships in the planning stages. Regional Turning Point field consultants are assisting partnerships in identifying local health improvement priorities, implementing local interventions, and evaluating impacts. Financial and technical resources are being secured from numerous collaborative resources to ensure the sustainability of the partnerships.

Turning Point continues to tailor public health needs in Oklahoma based on the real, perceived needs of community members who have joined public health officials as equal partners in making public health decisions. The Turning Point philosophy of community health improvement through collaborative state and local efforts has taken root in Oklahoma and is now built into the organizational fabric of the OSDH. Not only are the community Turning Point partnerships thriving but services and divisions within the OSDH seek ways to collaborate with Turning Point. In addition, other agencies and organizations outside of the OSDH are very much aware of Turning Point and frequently refer to the community Turning Point partnerships for ways to accomplish their own organizational goals within local communities. Turning Point has transformed public health in Oklahoma, and health status indicators in Oklahoma are beginning to show improvement. Because of Oklahoma's Turning Point initiative, the centralized public health system is reorganizing itself to take the following steps:

- Accept recommendations from stakeholder groups and coordinate untapped expertise among physicians and other health professionals, businesses, education, public health agencies, citizen groups, and the faith community
- Share responsibility for a community's health
- Find ways to share resources among agencies at the state and local levels
- Use available public health resources differently and with greater flexibility at the local level
- Accept accountability for the outcomes of public health decisions at both the state and local levels

These steps — which may appear to be fundamental and obvious — represent an extraordinary system change for Oklahoma. For the first time, communities have an equal voice in public health decisions. For the first time, public health workers within the OSDH see their role as supportive to community-based decisions and initiatives. And for the first time, community members see the important role they play in ensuring a healthier state for future generations. The results have been astounding, with numerous health education initiatives and sustained community system changes (6), including the following:

- Removal of sugar drinks and unhealthy snacks from school vending machines
- Passage of local health and safety ordinances
- Establishment of community health centers
- Founding of a new county health department and development of another
- Development of community trails for exercise
- Adoption of exercise and healthy eating by thousands of Oklahomans through Turning Point's *Walk This Weigh* campaign
- Enhancement of substance abuse and tobacco use prevention efforts

In addition to the health education initiatives and system changes occurring at the local level, numerous changes are happening statewide, including the following:

### Oklahoma Task Force on Health Disparities

This legislative task force is an outgrowth of Turning Point's effort to impact health outcomes by reducing health disparities (7).

### Oklahoma Certified Healthy Business Program

To date, 120 Oklahoma businesses have been certified as healthy by providing wellness opportunities for their employees. As a subcommittee of the statewide Oklahoma Turning Point Council, key partners include the Oklahoma State Chamber of Commerce and the Oklahoma Academy for State Goals (8).

### Public Health Institute of Oklahoma

An outgrowth of the Oklahoma Turning Point Council, the Public Health Institute of Oklahoma was created in early 2003 to be a neutral public health organization promoting positive health practices through collaboration among government, academia, and communities. The institute will 1) develop and expand funding resources for public health improvement projects; 2) build and develop community assets for health improvement, including leadership skills; 3) increase public health communication and networking; 4) advocate for core public health functions; 5) assist in reducing health disparities; and 6) evaluate components of local communities and the public health system (9).

## Final thoughts

The health education initiatives and community system changes in Oklahoma did not happen randomly. It took people who were not afraid to redefine their relationships with each other. Key leaders in Oklahoma's counties and at the OSDH were committed to spending the time necessary to build relationships and think differently about how to approach public health. Now, it's not about the "state" people or the "local" people. It's about *us*, working together to build healthy communities.

Turning Point will continue in Oklahoma. Organizationally, Turning Point is a division within the OSDH, under the Community Development Service. Plans are underway to hire additional Turning Point staff to support the efforts of Oklahoma's current Turning Point partnerships and expand into additional counties. The expanding activities of Turning Point also include work with faith-based organizations to eliminate health disparities and increase access to primary care services. Turning Point will continue to play a critical role in health improvement efforts in Oklahoma, both inside the OSDH and alongside many other agencies, organizations, and individual partners who have been part of Turning Point since 1997.

Perhaps the impact of Turning Point was best described by Ed Kirtley, past chairman of the Texas County Turning Point initiative:

Undoubtedly, the most important personal change from Turning Point is a better understanding of my community. . . . [M]y involvement in Turning Point created a new enthusiasm for public health and the potential for making an impact. I felt empowered to really create change — something that without the synergy of the group I would not have thought possible to do. Turning Point taught each of us that we can change and can more effectively serve our community if priorities and solutions are developed and implemented locally (10).
